# Use of cholic acid in Smith-Lemli-Opitz syndrome (SLOS): real-world patient outcomes

**DOI:** 10.1186/s13023-025-03914-x

**Published:** 2025-07-28

**Authors:** Edwin Ferren, Paul R. Hillman, Amy Kritzer, Joseph Ray, Alvaro Serrano, Hope Northrup, Paige Roberts, Rana Dutta, Tiziano Pramparo, Pamela Vig, Robert D. Steiner

**Affiliations:** 1 Department of Genetics, Atrium Health Levine Children’s Hospital, 1001 Blythe Blvd, Charlotte, NC USA; 2 Department of Pediatrics, Division of Medical Genetics, McGovern Medical School at UTHealth Houston, Houston, TX USA; 3 Boston Children’s Hospital, Boston, MA USA; 4 University of Texas Medical Branch, Houston, TX USA; 5 East Tennessee State University, Johnson City, TN USA; 6 Mirum Pharmaceuticals, Inc., Foster City, CA USA; 7 University of Wisconsin School of Medicine and Public Health, Madison, WI USA

**Keywords:** Cholic acid, Smith-Lemli-Opitz syndrome, SLOS, RSH syndrome, Cholesterol, Cholesterol deficiency, 7-dehydrocholesterol reductase (DHCR7), 7-dehydrocholesterol, 7-DHC, 8-dehydrocholesterol, 8-DHC

## Abstract

**Background:**

Smith-Lemli-Opitz Syndrome (SLOS) is an autosomal recessive disorder of cholesterol biosynthesis caused by biallelic pathogenic variants in *DHCR7*, which encodes the enzyme 7-dehydrocholesterol reductase (DHCR7). SLOS is a multisystemic disorder affecting various aspects of health, including growth, development, behavior, and quality of life, underscoring the need for safe, efficacious interventions that limit disease burden. DHCR7 enzyme deficiency leads to a “metabolic block” resulting in decreased cholesterol production and accumulation of its precursor 7-dehydrocholesterol and the secondary isomer 8-dehydrocholesterol. Reduced cholesterol synthesis, in turn, leads to decreased levels of cholic acid (CA), an endogenous bile acid synthesized from cholesterol and essential for cholesterol absorption. Dietary cholesterol supplementation is standard therapy. Bile acid supplementation with CA has been shown to improve dietary cholesterol absorption and raise plasma cholesterol levels. However, there is a paucity of patient-level data regarding the utility of CA as a treatment for SLOS. The purpose of this case series is to address the lack of comprehensive patient data through documentation of the outcomes of a company-sponsored CA patient experience program. A retrospective chart review was conducted for these individuals while on CA plus cholesterol supplementation. Data for demographics and key clinical/laboratory parameters were captured with a standardized data collection tool.

**Results:**

Eight genetically confirmed individuals with SLOS (age range 1 to 20 years) with median plasma cholesterol levels at baseline ≤ 125 mg/dL were treated with CA at 10–15 mg/kg/d for 30 to 450 days. Exogenous CA administration improved cholesterol levels in the majority of patients. Growth improved after CA initiation and trended toward age-appropriate growth percentiles. Reports from the patient, parent/caregiver, and/or healthcare professional noted positive behavioral changes leading to increased social interaction, cognitive engagement, and improved communication skills. Improvements in biochemical parameters and quality of life were also observed in several patients after CA treatment. CA supplementation was well tolerated with minimal adverse events.

**Conclusions:**

The cumulative experiences of eight patients provide a compelling narrative supporting the potential utility of CA treatment in SLOS while underscoring the safety of CA in this patient population. Larger longitudinal studies of CA in patients with SLOS are warranted.

## Background

Smith-Lemli-Opitz syndrome (SLOS) is an autosomal recessive disorder caused by biallelic pathogenic variants in *DHCR7* (HGNC:2860), which encodes 7-dehydrocholesterol reductase (DHCR7), the enzyme responsible for the last step in cholesterol biosynthesis [1–3]. 7-Dehydrocholesterol reductase enzyme deficiency in patients with SLOS leads to reduced levels of plasma cholesterol as well as an increase in plasma concentrations of the cholesterol precursor 7-dehydrocholesterol (7-DHC; cholesta-5,7-dien-3β-ol) and its isomer 8-dehydrocholesterol (8-DHC; cholesta-5,8-dien-3β-ol) [1, 4–6]. Reduced production of bile acids has been observed in patients with SLOS, which can also limit absorption of dietary cholesterol [7]. Smith-Lemli-Opitz syndrome presents with a spectrum of clinical manifestations of varying severity [8, 9], including multiple internal and external congenital malformations, developmental delays, restriction of growth, intellectual disabilities, aberrant behavior, and abnormalities in sterol synthesis [5, 8, 10, 11].

Common physical features of SLOS include microcephaly, 2–3 syndactyly of the toes, ptosis, anteverted nares, broad nasal bridge, and low-set ears [5, 9]. Behavioral issues have been reported in approximately 90% of patients, with elevated frequency of self-injurious behavior including self-biting, head banging, and a distinct forceful, rapid, arched backward diving motion of the upper body (opisthokinesis) [12]. Other reported behavioral manifestations include irritability, inconsolable screaming, hyperreactivity to visual and auditory stimuli, sleep disturbance, and social and communication deficits. Over half of patients meet diagnostic criteria for autism spectrum disorder (ASD) [11–14]. Poor feeding is common and leads to growth faltering, with many children requiring a gastrostomy tube (G-tube) for feeding [5, 15]. The clinical manifestations of SLOS have a profound adverse impact on quality of life in patients and caregivers [9, 14].

The estimated incidence of SLOS has been reported as approximately 1 in 20,000 live births in Europe and North America [16–20], with an estimated global prevalence of approximately 1 in 9437 individuals [21]. An estimated incidence of approximately 1 in 1590 to 1 in 13,500 has been reported based on calculated carrier frequency for all genetic variants [17]. The apparent disparity between observed incidence of the disorder and estimated incidence based on carrier frequencies may be due to factors such as in utero lethality, postnatal death before diagnosis, failure to diagnose milder cases, and misdiagnosis of severe cases [17, 22, 23].

Although the diverse disease-related manifestations in patients with SLOS are driven by disruption of key physiologic functions due to cholesterol deficiency, increased production of cholesterol precursors has also been proposed to play an important role in the pathophysiology of SLOS in part via oxidation to neurotoxic sterols [1, 7]. The goals of treatment for SLOS are aimed at increasing cholesterol levels and decreasing the levels of potentially toxic 7-DHC and 8-DHC. Clinical goals focus on improving developmental and behavioral problems such as delays in achievement of developmental milestones, irritability, self-injurious behavior, and sleep difficulties. Use of dietary cholesterol supplements to raise cholesterol levels is the current mainstay of treatment [14]. However, only a few well-controlled clinical trials have been published, none of them showing a clear benefit of cholesterol supplementation on development and behavior [24, 25]. A number of different treatment approaches, such as blocking the cholesterol biosynthesis pathway and increasing absorption of dietary cholesterol, have been proposed with the goal of decreasing the levels of 7-DHC and 8-DHC and/or increasing cholesterol levels to prevent the long-term consequences of the disorder.

The effects of the combination of cholesterol supplementation with β-hydroxy-β-methylglutaryl coenzyme A (HMG-CoA) reductase inhibitors (statins) have been tested as a means of mitigating the elevations of 7-DHC and 8-DHC in patients with SLOS [26, 27]. There is limited high-quality evidence supporting statin use in SLOS, and further studies will need to be conducted in a real-world setting. However, the rationale of statin use in SLOS is to inhibit HMG-CoA reductase, thereby blocking the cholesterol biosynthesis pathway upstream of the defect in DHCR7. This results in a reduction of both toxic precursors, 7-DHC and 8-DHC, while reducing the serum and cerebrospinal fluid concentrations of 7-DHC [26, 28].

Bile acids facilitate the digestion and absorption of fats and fat-soluble vitamins in the intestine. Cholic acid (CA) is approved for treatment of bile synthesis disorders caused by single enzyme defects [29]. The mechanism of action of CA has not been fully elucidated. However, patients with SLOS have a defect in cholesterol production and cholesterol is the precursor to the formation of primary endogenous bile acids (ie, CA). Patients with SLOS exhibit reduced cholesterol synthesis, and therefore, reduced synthesis of CA, which likely leads to a deficiency of CA. Cholic acid, a bile acid, is needed for absorption of fats and fat-soluble vitamins, nutrients, and optimal cholesterol absorption. In a small pilot study of 12 participants with SLOS and baseline plasma cholesterol levels ≤ 125 mg/dL, treatment with CA at 10 mg/kg/d twice daily for 2 months resulted in increases in plasma cholesterol levels in 11 participants ranging between 3.8 and 85.7% (mean 38.7%), and no significant changes in 7-DHC or 8-DHC levels were observed [7].

We present a case series of eight patients with SLOS who were treated with CA plus cholesterol supplementation in a real-world setting for up to 450 days and report on clinical findings of treatment effectiveness, including improvements in laboratory measures (eg, changes in plasma levels of cholesterol and precursors) as well as clinical outcomes (development and behavior).

## Methods

This series involved retrospective chart review for patients treated with CA plus cholesterol supplementation as part of a company-sponsored CA patient experience program. Information regarding individuals diagnosed with SLOS who were actively receiving treatment or had completed treatment was solicited from the treating physicians via standardized case report forms. Details collected included a summary of unique patient presentation, baseline characteristics of each patient, genetic information, cholesterol supplementation, intervention history, behavioral profile, and quality of life (QoL) information. The case report form captured quantitative changes in anthropometric measures (height, weight, growth) and laboratory values (levels of plasma total cholesterol, plasma 7-DHC, plasma 8-DHC, liver enzymes). Additionally, qualitative assessments of patient status per parental report, physician observation, and/or unsolicited teacher correspondence (eg, QoL, behavioral, cognitive, other), as well as information on duration of treatment, medication compliance, and adverse events (AEs) following initiation of treatment with CA, were collected.

## Results

Eight patient cases were reviewed. The baseline characteristics of each patient are summarized in Table 1. Five patients were male, and three were female (Table 1). The duration of treatment with CA ranged from 30 to 450 days*.*

**Table 1:** Baseline characteristics of cases before treatment with cholic acid

	Patient 1	Patient 2	Patient 3	Patient 4	Patient 5	Patient 6	Patient 7	Patient 8
Age	6 years	4.7 years	1 year	NR	7 years	20 years	5 years	5 years
Sex	Male	Female	Female	Male	Male	Female	Male	Male
Age at diagnosis	4 years	23 months	8 days	2 days	Shortly after birth	6 weeks	1–2 days	~ 1 year
*DHCR7* variants	c.1349_1350delinsTG c.964-1G > C	c.964-1G > C c.546G > T	c.724C > T c.770C > G	NR	2.3-kb deletion^a^ c.278C > T	c.452 G > A c.296 T > C	c.906C > G c.964 + 1G > C	c.452G > A c.1054C > T
7-DHC, mg/dL	5.5	9.1	0.81	5.4	8.1	28.62	32.51	20.85
8-DHC, mg/dL	4.7	7.3	0.62	NR	NR	7.36	7.21	5.76
Total cholesterol, mg/dL	139	116	107	103	50	115	31.7	26
(7-DHC + 8-DHC) to total cholesterol ratio	0.073	0.141	0.013	NR	NR	0.313	1.253	1.023
AST, U/L	38	36	13	50	NR	31	47	34
ALT, U/L	30	15	19	40	NR	43	27	26
Height Z-score	− 1.33	− 3.31	− 1.28	− 1.74	NR	− 2.84	− 4.71	NR
Weight Z-score	− 1.65	− 5.92	− 1.92	− 2.6	NR	− 5.54	− 2.65	NR
Requirement for G-tube	No	Yes	Yes	Yes	Yes	Yes	Yes	Yes
ASD diagnosis	Yes	NR	Pending	No	Yes	Not done	No	NR
Sleep disturbance	No	Yes	No	No	Yes	NR	Yes	NR
Self-injurious behavior	No	NR	No	No	NR	Head banging	Occasional finger biting	NR

ALT, Alanine aminotransferase; ASD, Autism spectrum disorder; AST, Aspartate aminotransferase; DHC, Dehydrocholesterol; *DHCR7*, 7-dehydrocholesterol reductase gene; G-tube, gastrostomy tube; NR, Not reported

^a^Deletion of exon 9 with breakpoints in intron 8 and postcoding regions

**Fig. 1:**
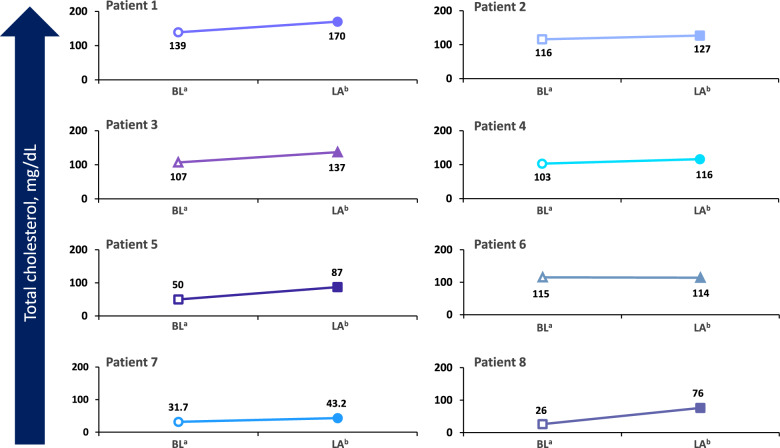
Total cholesterol levels over time during treatment with cholic acid. BL, baseline; LA, last assessment during treatment with cholic acid. ^a^Baseline for each patient was defined as Day 0. ^b^Time of last cholesterol assessment for each patient was: Patient 1 (Day 450); Patient 2 (Day 450); Patient 3 (Day 213); Patient 4 (Day 150); Patient 5 (Day 30); Patient 6 (Day 30); Patient 7 (Day 180); Patient 8 (Day 365)

**Fig. 2:**
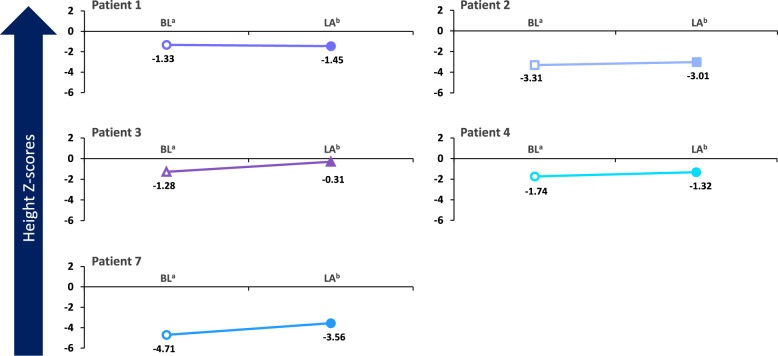
Change in height Z-scores over time during treatment with cholic acid. BL, baseline; LA, last assessment during treatment with cholic acid. ^a^Baseline for each patient was defined as Day 0. ^b^Time of last height assessment during treatment for each patient was: Patient 1 (Day 450); Patient 2 (Day 450); Patient 3 (Day 213); Patient 4 (Day 150); Patient 7 (Day 120)

**Fig. 3:**
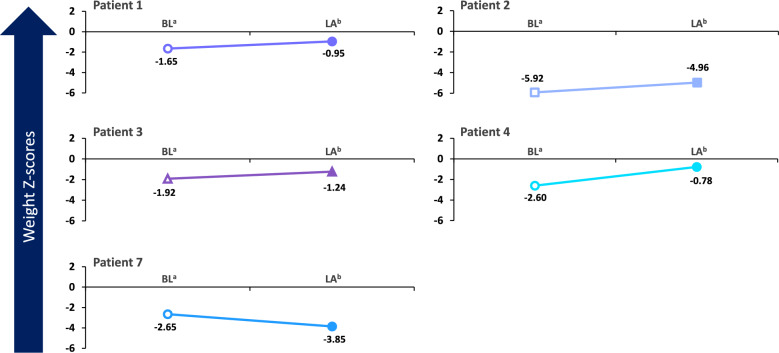
Change in weight Z-scores over time during treatment with cholic acid. BL, baseline; LA, last assessment during treatment with cholic acid. ^a^Baseline for each patient was defined as Day 0. ^b^Time of last weight assessment during treatment for each patient was: Patient 1 (Day 450); Patient 2 (Day 450); Patient 3 (Day 213); Patient 4 (Day 150); Patient 7 (Day 120)

Full vignettes for each case are presented below. Clinically meaningful qualitative improvements observed during treatment with CA are summarized in Table 2. Results for baseline and posttreatment quantitative laboratory assessments for cholesterol are shown in Fig. 1. Estimated height and weight Z-scores at baseline and posttreatment are shown in Figs. 2 and 3, respectively. Results of additional laboratory assessments as well as behavioral, developmental, and QoL measures are described in each vignette.

**Table 2:** Qualitative improvements observed during treatment with cholic acid

	Patient 1	Patient 2	Patient 3	Patient 4	Patient 5	Patient 6	Patient 7	Patient 8
QoL changes	Yes – more social and interactive	Yes – more interactive and playful	NR	Yes – more interactive	Yes – happier; part of adaptive sports program	Yes – more alert, expressive, and interactive; improved mobility	Yes – better	Yes – better
Behavioral changes	Positive	Positive	NA (patient aged 12 months)	Positive	Positive	Positive	NR	Positive. Favorable behavioral response noted
Improved speech	NR	Yes	NR	Yes	Yes	NR	NR	Yes – more vocal and using words appropriately
Cognitive and developmental improvements	NR	Yes –progressing with milestones and now able to walk unassisted	Yes – developmental milestones met on time (crawling, pulling to stand)	NR	Yes – slowly gaining developmental skills	Yes – able to make choices and communicate needs (making clear choices when expressing needs and wants); improved mobility	Yes	Yes – progress in milestones has been observed; started prekindergarten home program and attends school 2 days per week; able to walk with support

NA, Not applicable; NR, not reported; QoL, Quality of life

### Case 1

Patient 1 is a 6-year-old male who was noted to have flattened facial features, anteverted nares, and 2–3 syndactyly of the toes at birth. At 4 years of age, he was referred for genetic testing due to concerns for ASD, developmental delays, and microcephaly. The initial diagnosis of SLOS was based on elevated 7-DHC levels (6.8 mg/dL, normal range 0.004–0.036 mg/dL) and confirmed by genetic testing, which showed compound heterozygosity for two pathogenic variants in *DHCR7,* [c.1349_1350delinsTG] and [c.964-1G > C]. The child did not require a G-tube but had feeding problems, with subsequent growth faltering. He began cyproheptadine (Periactin) with limited benefit in weight gain and was referred to the feeding clinic. His baseline growth parameters showed a height Z-score of − 1.33 and weight Z-score of − 1.65. Before treatment with CA, he received cholesterol supplementation at 50 mg/kg for 7 months, with some improvements noted in behaviors at home and school.

#### Early development

The patient smiled at 3 months of age, reached for objects at 3 to 4 months, rolled over at 4 months, and transferred objects at 4 to 5 months. At 5 to 6 months, he was able to sit with support and sat alone at 7 months. He crawled at 9 months, walked with assistance at 10–11 months, and walked alone at 15 months. At 18 months, he spoke his first words, then began making sentences at 3 to 3.5 years. He was toilet trained at the age of 3.5 years. He slowly progressed with language and cognitive development. He was diagnosed with a nonspecific neurodevelopmental disorder and was determined to have a Full-Scale Intelligence Quotient of 75. Delayed social and emotional development were noted. Atypical behaviors common to children with ASD were observed, including delayed inconsistent imitation, behavioral rigidity and difficulty with change in transition, perseverative interest in playing, inconsistent visual response, inconsistent response to name, and some motor stereotypy. The patient had no history of sleep problems or self-injurious behavior. He had a normal neurologic examination; although his small head size was noted, it was considered proportionate to his body size. He was seen by neurosurgery due to microcephaly and a prominent metopic ridge, but no intervention was performed. Ear tubes were inserted due to frequent ear infections.

#### Outcome with CA treatment

The patient had a baseline cholesterol level of 139 mg/dL (normal range ≤ 170 mg/dL in children, ≤ 200 mg/dL in adults) [30], 7-DHC level of 5.5 mg/dL, 8-DHC level of 4.7 mg/dL (normal range ≤ 0.03 mg/dL) [31], and 7-DHC + 8-DHC to cholesterol ratio of 0.073 while on cholesterol supplementation at 50 mg/kg/d. After adding CA treatment at 10 mg/kg/d, total cholesterol level improved to 170 mg/dL at the patient’s last visit on Day 450, 7-DHC level declined to 3.6 mg/dL, 8-DHC level declined to 4.0 mg/dL, and 7-DHC + 8-DHC to cholesterol ratio decreased to 0.045. Baseline and Day 450 liver enzyme values, respectively, were 30 and 22 U/L for alanine aminotransferase (ALT) and 38 and 36 U/L for aspartate aminotransferase (AST). Behavioral and developmental outcomes following treatment with CA included continued improved behavior at school; enhanced social interactions were noted after 60 days of treatment with CA and continued to improve up to Day 450. The child is very interactive and doing well in school, with a decline in negative behaviors. He continues to do well on 10 mg/kg/d of CA, demonstrating marked improvements following treatment with the medication. Before starting CA, his weight was in the 5th percentile, improving substantially after treatment to the 17th percentile. At Day 450, his height Z-score was –1.45, and his weight Z-score was –0.95. Additionally, the patient’s head circumference Z-score was less than –2.05, and his body mass index (BMI) Z-score was + 0.13.

### Case 2

Patient 2 is a female referred for genetic testing at 23 months of age due to poor weight gain, short stature, microcephaly, anteverted nares, a flattened facial profile, retrognathia, 2–3 syndactyly of the toes, cleft palate, generalized hypotonia, and concerns of Pierre Robin sequence. Intracranial ventriculomegaly was noted on prenatal ultrasounds and led to initial concerns. A diagnosis of SLOS was confirmed with a 7-DHC level of 10.4 mg/dL (normal range 0.004–0.036 mg/dL). Genetic testing showed compound heterozygosity for a pathogenic *DHCR7* variant, [c.964-1G > C], and a likely pathogenic variant, [c.546G > T]. At initial assessment (23 months of age), the patient weighed 7 kg (Z-score − 4.16) and was 0.71 m in height (Z-score − 4.52), with a head circumference Z-score of − 2.20 and a BMI Z-score of − 1.3. At baseline (29 months), her height (Z-score − 3.31) and weight (Z-score − 5.92) were below the mean for her age. Her head circumference Z-score was − 2.79, and her BMI Z-score was − 3.26. A G-tube was placed due to a poor suck response and feeding difficulties. The patient was initially evaluated by an endocrinologist for short stature and poor weight gain and then referred for genetic testing for further evaluation. The patient was also seen by an audiologist, who recommended ear tubes due to infections. Cholesterol supplementation was started 6 months before CA treatment and continued at 50 mg/kg/d through Day 450. The patient received surgery for cleft palate repair. Magnetic resonance imaging at 6 months showed mild, stable ventriculomegaly. At the last follow-up, she continued to be monitored by neurosurgery for ventriculomegaly and microcephaly.

#### Early development

Initial developmental concerns were noted when the patient was not crawling. Some improvements in behavior and weight were observed around 6 months of age. The patient sat with help at the age of 6 months and unassisted at 10 months. She crawled at 11 months. At 23 months, she was able to take steps while holding onto her parent’s hands but was not yet walking independently. She could say a few single words but did not put words together, and her speech was difficult to understand. At the last follow-up, she was learning sign language. She is a poor sleeper but has no history of self-injury.

#### Outcome with CA treatment

The patient began CA treatment at 15 mg/kg/d (100 mg). Laboratory findings following initiation of treatment included a slight increase in total cholesterol level from 116 mg/dL at baseline to 127 mg/dL at last visit on Day 450 (normal range ≤ 170 mg/dL in children, ≤ 200 mg/dL in adults) [30]. In addition, increases from baseline to Day 450 were observed in 7-DHC level (9.1–11.3 mg/dL), 8-DHC level (7.3–10.1 mg/dL (normal range ≤ 0.03 mg/dL) [31], and 7-DHC + 8-DHC to cholesterol ratio (0.141–0.169). The patient’s levels have fluctuated during treatment. Her baseline and Day 450 liver enzyme values, respectively, were 15 and 19 U/L for ALT and 36 and 36 U/L for AST. Behavioral improvements in addition to weight gain were observed starting around 6 months of age. Her weight slowly improved, although she did have some setbacks with illnesses. She began walking with assistance around 6 to 8 months after initiation of therapy and walked independently 1 year after treatment initiation. Since initiation of CA, the patient has learned more words, has been able to sign a few words, has shown much more interactivity and playfulness, and has had improved sleep. At the time of last treatment, the patient had a height Z-score of − 3.01, weight Z-score of − 4.96, head circumference Z-score of − 3.96, and BMI Z-score of − 3.45. Her cholesterol supplementation was increased to 80 mg/kg/d in attempts to further improve cholesterol level and decrease her 7-DHC level.

### Case 3

Patient 3 is a female presenting at birth with unilateral cleft hard palate, patent ductus arteriosus (PDA), ventricular septal defect, atrial septal defect, pulmonary hypertension, hypotension, and bilateral 2–3 syndactyly of the toes. Shortly after birth, the patient required intensive care for feeding issues, including G-tube placement, and life support due to hypotension and hypoxia. Dysmorphic features included micrognathia, hypertelorism, and possible craniosynostosis, which, along with syndactyly, raised the suspicion of SLOS and prompted genetic and biochemical testing. Family history included a paternal cousin with congenital lipomatous overgrowth; progressive, complex, and mixed truncal vascular malformations; epidermal nevi; and skeletal anomalies (CLOVES syndrome). Genetic testing revealed biallelic pathogenic variants in the *DHCR7* gene, [c.724C > T] and [c.770C > G]. Nutritional supplements before the administration of CA at 1 month included synthetic cholesterol (Cholextra) at 300 mg/d (88 mg/kg/d), elemental formula (Elecare), and expressed breast milk (EBM) at approximately 100 kcal/kg/d.

#### Early development

The patient met developmental milestones. She was able to control her head movements at 2 to 3 months of age, rolled over at 5 months, sat at 5 months, crawled at 5 months, and pulled herself up to a standing position at 10 months. Patient was walking unassisted at 12 months of age. She remained dependent on G-tube feeding. No behavioral issues had appeared by 12 months of age. No IQ testing or formal evaluation for ASD had been performed due to the patient’s age.

#### Outcome with CA treatment

Starting at 1 month of age, the patient received CA at 11 to 14 mg/kg/d for 11 months. At 7 months of treatment, laboratory findings showed change in total cholesterol level from 107 mg/dL (baseline) to 137 mg/dL (normal range ≤ 170 mg/dL in children, ≤ 200 mg/dL in adults) [30], a decrease in 7-DHC level from 0.81 mg/dL (baseline) to 0.69 mg/dL (normal range ≤ 0.2 mg/dL) [31], and an increase in 8-DHC level from 0.62 mg/dL (baseline) to 1.28 mg/dL (normal range ≤ 0.03 mg/dL) [31]. The patient’s liver enzyme values were stable at 19–20 U/L for ALT and increased from 13 to 30 U/L for AST. Her growth has been acceptable (weight, 2.74–9.84 percentile; height, 10.3–49.6 percentile; fronto-occipital circumference [FOC] relatively unchanged at 0.6–0.41 percentile per the female World Health Organization growth chart), and she met more milestones than expected. During treatment, her height and weight Z-scores have improved from − 1.28 and − 1.92 at baseline to − 0.31 and − 1.24 at 7 months, respectively. Due to her age of 1 month when she began CA supplementation, changes in behavior with treatment have not been assessed. The dose of CA has been increased with the child’s growth and kept between 10 and 15 mg/kg/d. One AE was reported—blue/purple toes and fingers, which typically occurred in the morning—1 week after increasing the CA dose, but no changes were made to her treatment. Her compliance is good, without dose interruptions, dose reductions, or recurrence of digit color changes. Currently, the child is 12 months of age, with a weight of 7.71 kg and height of 74.3 cm. She is receiving speech, occupational, and physical therapy.

### Case 4

Patient 4 is a male who presented with dysmorphic facies (microcephaly, microretrognathia, epicanthal folds, low-set ears, upturned nose, anteverted nares, and downturned mouth), polydactyly, syndactyly, and hypospadias. He had poor feeding, a congenital heart defect, and difficulty breathing at 2 days of life, requiring intensive care. The suspicion of SLOS was confirmed by low cholesterol level of 76 mg/dL (normal range ≤ 170 mg/dL in children, ≤ 200 mg/dL in adults), [30] and elevated 7-DHC level of 5.4 mg/dL (normal range ≤ 0.2 mg/dL) [31]. Conventional and molecular karyotyping results were normal, and *DHCR7* gene sequencing results are unavailable. Family history is noncontributory. The patient received synthetic cholesterol supplementation (cholesterol NF) at 150 mg/d via G-tube as an infant and then transitioned to 3 egg yolks per day and 6 cartons of Kate Farms Pediatric Standard supplemental formula (1.2 kcal/mL) daily.

#### Early development

At 2 days of life, the patient was 45.5 cm in length (3rd percentile), weighed 2810 g (10th percentile), and had FOC of 32 cm (< 3rd percentile). Soon after birth, he was evaluated by neonatal intensive care unit (NICU) specialists, cardiology, surgery, and urology. While in the NICU, a G-tube was placed and a Nissen fundoplication was performed. Subsequently, he had hypospadias repair and polydactyly excision. The boy is high functioning and is able to dress and feed himself and communicate his desires to his family. No formal IQ testing has been conducted. At the time of writing, he was performing well in a 3rd-grade special education class and spoke in 3- to 4-word sentences in both English and Spanish. He had no concerning behavioral anomalies, got along well with his siblings and classmates, and did not fit the diagnostic criteria for ASD.

#### Outcome with CA treatment

Laboratory analyses revealed an increase in total cholesterol level from 103 mg/dL at baseline to 116 mg/dL after 150 days of CA treatment. His liver enzyme values decreased from 40 U/L to 24 U/L for ALT and increased from 50 U/L to 56 U/L for AST. Since starting CA, the child’s height and weight Z-scores have improved substantially, increasing from –1.74 and –2.6 at baseline to –1.32 and –0.78 at 150 days, respectively. At the last assessment, his height and weight were within the normal Centers for Disease Control (CDC) growth curves for the first time in his life (5th and 4th percentile, respectively). His BMI was at the 14th percentile. Behavioral and developmental changes included improvement in appetite by Day 150 of CA treatment, and he was more interactive and performing better in school. His speech was more coherent, and he began helping his younger sister with Spanish to English translation. Compliance with the medication is good, and no AEs have been reported.

### Case 5

Patient 5 is a male who presented at birth with multiple congenital anomalies, including microcephaly, microphallus with hypospadias, bilateral 2–3 syndactyly of the toes, single palmar creases, and cleft soft palate with a small extension into the hard palate. Laboratory testing showed a low cholesterol level of 50 mg/dL, and the patient was started on cholesterol supplementation at 250 mg/kg/d at 2 weeks of life. Genetic testing showed compound heterozygosity for *DHCR7* pathogenic variants, identifying a 2.3-kb deletion that included all of exon 9 with breakpoints in intron 8 and postcoding regions, and a missense variant, [c.278C > T]. The patient has undergone several procedures, including bilateral myringotomy with ventilation tube placement, palatoplasty, supraglottoplasty, adenoidectomy, bilateral tympanostomy tube placement, lingual frenotomy, and hypospadias repair. He receives additional medications, mostly for allergies, including albuterol, fluticasone propionate (Flonase), clonidine, and hydrocortisone as needed. He is fed through a G-tube and consistently receives 1 pouch of Nourish 5 times daily as well as cholesterol supplementation at 180 mg/kg/d. He also takes some foods by mouth.

#### Early development

The patient experienced developmental delay with poor growth, frequent vomiting, and reflux. At 7 years of age, his performance indicated a developmental level of less than 2 years of age (Bayley-III Cognitive age equivalent = 15 months); however, his functioning as reported by his mother is like that of a 3- or 4-year-old child. He is perceptive of his environment and can walk without a walker, jump, and climb. He is beginning to mimic, but he needs help with all activities of daily living. He knows several words and is beginning to speak more and combine words. The boy exhibits anxiety and irritability and is a poor sleeper. He was diagnosed with ASD and has moderate intellectual disability.

#### Outcome with CA treatment

Laboratory findings included an increase in total cholesterol level from 50 mg/dL at baseline to 87 mg/dL (normal range ≤ 170 mg/dL in children, ≤ 200 mg/dL in adults) [30], and a slight increase in his 7-DHC level from 8.1 mg/dL at baseline to 9.3 mg/dL (normal range ≤ 0.2 mg/dL) after CA administration [31]. The family reported improvements in behavior, constipation, speech skills, and overall happiness after beginning CA treatment. At the time of this writing, the patient is thriving in a special education program, participating in an adaptive sports program, and slowly gaining in developmental skills. The patient is receiving 10 mg/kg/d of CA and has remained on this treatment for more than 1 year with good compliance; however, treatment was briefly interrupted due to prior authorization. He experienced an AE of elevated liver enzymes 1 year ago but has not experienced any other events.

### Case 6

Patient 6 is a female who was diagnosed at another institution but has been followed at her current clinic since the age of 3 years. She exhibits microcephaly, cleft palate, a short mandible with prognathism, smooth philtrum, poor dentition with malalignment, polydactyly, syndactyly, PDA, patent foramen ovale, contractures at the knees, scoliosis, and severe developmental delay; in addition, she met requirements for a G-tube. Genetic testing revealed heterozygosity for pathogenic *DHCR7* variants, [c.452G > A] and [c.296T > C]. Before initiation of CA treatment, the patient was 20 years of age, weighed 33.1 kg, and was 144.8 cm in height. Her FOC was 49 cm. Her weight, height, and FOC Z-scores were − 5.54, − 2.84, and − 2.05, respectively. Surgical interventions have included cleft palate repair and G-tube placement. Medications before initiation of CA included lamotrigine (Lamictal), montelukast, norgestimate-ethinyl estradiol, and ursodiol. The patient’s nutrition is managed via a G-tube with hypoallergenic infant formula (Neocate) due to suspected corn allergy and commercial dried egg yolk as bolus feeds 4 times per day to provide approximately 30 kcal/kg/d and 1.2 g/kg/d of protein. The patient will take occasional tastes of food by mouth, but not reliably. Before starting CA, she received cholesterol supplementation at 89 mg/kg/d (1939 mg daily) from 3 years of age. The patient previously participated in a SLOS clinical trial which involved cholesterol supplementation.

#### Early development

The patient is totally dependent for activities of daily living and is nonverbal but generally cooperative and sociable. She has a history of head banging, but no testing for ASD or IQ has been performed. She receives physical, occupational, and speech therapy and uses orthotics (hand splints), a walker, and a wheelchair.

#### Outcome with CA treatment

The patient was already an adult when she initiated CA treatment. Therefore, the expectations were that the treatment may help her better absorb her cholesterol supplementation rather than lead to a drastic difference in her development or behavior. She has been treated with CA at 14 mg/kg/d (ie, 250 mg twice daily) for approximately 1 year. Compliance is good, and there have been no dose changes. However, after being on the medication for a few months, there was a gap due to an insurance requirement for genetic testing. Laboratory findings include no change in total cholesterol (115 mg/dL at baseline vs 114 mg/dL on Day 30; normal range ≤ 170 mg/dL in children, ≤ 200 mg/dL in adults) [30], as well as a slight increase in 7-DHC from 28.62 mg/dL at baseline to 31.35 mg/dL (normal range ≤ 0.2 mg/dL) [31] and an increase in 8-DHC from 7.36 mg/dL at baseline to 20.64 mg/dL at 10 months (normal range ≤ 0.03 mg/dL) [31]. The patient’s baseline and Month 10 liver enzyme values, respectively, were 43 and 15 U/L for ALT and 31 and 19 U/L for AST. While receiving CA, she showed improvement in cognitive status by being more interactive, alert, and expressive and making clear choices when expressing her needs and wants. Additionally, her physical skills improved such that she could wheel herself across the house in her wheelchair. During the gap in CA treatment, the family observed a significant decline in her behavior, but the improvements were restored once CA was restarted and maintained throughout treatment.

### Case 7

Patient 7 is a male diagnosed with SLOS within the first few days of life by a clinical geneticist. The initial diagnosis was made based on the presence dysmorphic features. He also had multiple congenital anomalies before and at birth, including intrauterine growth restriction, cleft palate, cardiac disease (a valve issue that later resolved), bilateral congenital ptosis, hypotonia, dysgenesis of the corpus callosum, oropharyngeal dysphagia, partial gastric outlet obstruction by an antropyloric channel abnormality (narrow and elongated with redundant circumferential folds), and bilateral 2–3 syndactyly of the toes. He also had a history of Pierre Robin sequence and was diagnosed with hypertension in infancy. The diagnosis of SLOS was confirmed in the first few days of life, with laboratory testing showing plasma 7-DHC level of 7.8 mg/dL (normal range 0.004–0.036 mg/dL) and total cholesterol level of 12 mg/dL (normal range ≤ 170 mg/dL in children, ≤ 200 mg/dL in adults [30]). Genetic testing showed heterozygosity for pathogenic *DHCR7* variants, [c.906C > G] and [c.964 + 1G > C]. The patient had undergone two surgeries: one for cleft palate and tongue and another to insert a G-tube. Before CA treatment, he received melatonin by enteral route and omeprazole 20-mg delayed release capsules. He presented with feeding challenges and a history of growth faltering. He is G-tube dependent for feeding due to oropharyngeal incoordination. He receives Nourish Peptide supplemented with 1 teaspoon of avocado oil and 2 hard-boiled egg yolks daily. Cholesterol supplementation with 1 egg yolk daily began in infancy, with dosing increases commensurate with weight. The patient benefited from occupational, physical, and speech therapy, which began in infancy, before attending a special needs school program a few hours each day. He continues to be followed by dietitians, neurology, plastic surgery, developmental pediatrics, clinical genetics, and pediatric gastrointestinal specialists. Since resolution of a thickened and domed pulmonary valve, he is no longer followed by cardiology.

#### Early development

The patient remained in the NICU for 2 months after birth due to respiratory and feeding issues and exhibited developmental delays. At the age of 3 years 1 month (before CA treatment), he was able to sit without support and rolled to move around a room but did not crawl. He did not stand or walk and had a poor protective response. He seemed to understand much of what was said to him and was very sociable. The child vocalizes frequently and keeps his mouth open much of the time. He puts his hands to his midline and crosses them and puts his hands to his mouth. He occasionally bites his fingers and sometimes throws himself backward when sitting, but he otherwise does not exhibit self-injurious or self-mutilative behavior. No head banging was observed. He has a sleep disorder but no obvious signs of ASD.

#### Outcome with CA treatment

Currently, the patient receives CA at 150 mg daily. His baseline cholesterol level was 31.7 mg/dL and increased to 43.2 mg/dL at Day 180. Additional laboratory findings included a decrease in 7-DHC level from 32.51 mg/dL at baseline to 17.52 mg/dL at Day 180 and an increase in 8-DHC level from 7.2 mg/dL at baseline to 12.5 mg/dL at Day 180 (normal range ≤ 0.03 mg/dL) [31]. His baseline and Day 180 liver enzyme values, respectively, were 27 and 36 U/L for ALT and 47 and 28 U/L for AST. His weight at baseline was 9.16 kg and increased to 11.1 kg after starting treatment. Notable behavioral and developmental changes were observed at the age of 4 years 5 months and after start of CA treatment, including the ability to sit up without support but not standing or walking without support. The patient uses a stander and gait trainer. He does not say words, is not signing, and does not seem to respond to commands, but he is able to communicate some of his needs. He is currently in school 3 h per day, which is going well, and he is taking swimming lessons. Since starting CA, his overall health and progress in development have been good. He is making slow gains across all domains and continues to benefit from speech, occupational, and physical therapy. At the time of this writing, the boy is 5 years of age with a weight of 11 kg and height of 0.883 m.

### Case 8

Patient 8 is a 5-year-old male in whom SLOS was suspected prenatally; it was postnatally confirmed with molecular diagnosis. He passed his newborn hearing test. The patient presented with SLOS-associated phenotype of microcephaly, hypotonia, left multicystic dysplastic kidney, cleft palate, adrenal insufficiency, 2–3 syndactyly, and hypogenitalism with hypospadias. He had COVID-19 infection at 36 months of age with no major complications and received stress dosing of corticosteroids. The diagnosis of SLOS was confirmed in the first year of life, with compound heterozygous pathogenic *DHCR7* variants, [c.452G > A] and [c.1054C > T], demonstrated via genetic analysis. Both parents are confirmed carriers. Surgical history included insertion of a G-tube at 1 month of age, repair of hypospadias, ear tube placement, and cleft palate repair. Treatments include cholesterol supplementation (95 mg/kg/d), coenzyme Q10, and hydrocortisone at 25 mg intravenously/intramuscularly before procedures. Constipation is managed with milk of magnesia rather than polyethylene glycol (MiraLAX). The patient was followed by multiple specialists, including a geneticist, SLOS specialist/researcher, urologist, endocrinologist, pulmonologist, and ophthalmologist (for a short interval).

#### Early development

The child presented with cognitive impairment, level 3 ASD, and mobility impairment, although he was able to cruise with assistance.

#### Outcome with CA treatment

The patient started on CA at 10 mg/kg/d, divided into 3 doses, at 50 months of age. Two months after initiating therapy, he presented with acute pancreatitis of unknown etiology, and CA was put on hold for 1 month. Clinical exome sequencing, including mitochondrial DNA analysis, was requested, confirming his primary diagnosis with no additional findings. He was restarted on CA at 10 mg/kg/d at 53 months of age and was brought back to a geneticist for a follow-up visit at 120 days. He has continued on CA with no other major concerns and continues to receive G-tube feeds with Nourish Peptide (210 mL) 6 times per day with no overnight feeds. Findings from laboratory assessments included an increase in total cholesterol level from 26 mg/dL at baseline to 76 mg/dL at 12 months (normal range ≤ 170 mg/dL in children, ≤ 200 mg/dL in adults) [30]. Additionally, at 12 months, 7-DHC level increased from 20.85 mg/dL at baseline to 30.8 mg/dL (normal range ≤ 0.2 mg/dL) [31], 8-DHC level increased from 5.76 mg/dL at baseline to 18.43 mg/dL (normal range ≤ 0.03 mg/dL) [31], and 7- DHC + 8-DHC to cholesterol ratio decreased from 1.023 at baseline to 0.167. Baseline and Day 365 liver enzyme values, respectively, were 26 and 63 U/L for ALT and 34 and 49 U/L for AST. After initiation of CA treatment, the patient became more vocal and used words appropriately. Additionally, he has been able to start a prekindergarten home program, attending school 2 mornings a week in a small class, and he has been enjoying this experience. At the time of this writing, he can walk with support using a walker and is able to have bowel movements in the toilet, but he still needs diapers for the entire day for urination. His height and head circumference have been at the 3rd percentile according to CDC growth curves, with improved weight and BMI over the last year and weight in the 10^th^–25th percentile.

## Discussion

Smith-Lemli-Opitz Syndrome is an inherited condition in which biallelic variants in *DHCR7* not only disrupt cholesterol biosynthesis and cholesterol production but also lead to overaccumulation of toxic cholesterol precursors, 7-DHC and 8-DHC, and various oxysterols derived from these precursors [5, 9, 14]. The central role of cholesterol in cellular signaling as an essential component of cellular membranes, as the signal for synapse formation, and as a key precursor in steroid hormone synthesis may help to explain the wide array of developmental defects and devastating physical, neurocognitive, and behavioral sequelae observed in patients with SLOS [5]. Notably, the clinical presentation of SLOS is highly variable in both the occurrence and severity of specific disease-related manifestations [8, 9, 32]. This variability in phenotypes is consistent with the identification of more than 100 different variants in the *DHCR7* gene, a key pathogenic event resulting in loss or abnormal enzymatic function of DHCR7. [5, 32]

Despite the identification of the key biochemical dysfunction involved in pathogenesis of the disease, current treatment approaches focusing on increasing cholesterol and/or decreasing levels of cholesterol precursor (7-DHC) and its derivative isomer (8-DHC) have demonstrated limited effectiveness [24, 25, 28, 33, 34]. There is no consensus regarding optimal therapy. Importantly, there is no clear evidence of long-term clinical improvement with these approaches. One key factor that may impact the effectiveness of cholesterol supplementation is the reduced production of bile acids in patients with SLOS, which has been linked to limited absorption of dietary cholesterol [7]. These findings provide a logical rationale for increasing bile acid levels as a therapeutic strategy that may lead to increased cholesterol absorption. Cholic acid is approved for treatment of bile acid synthesis disorders caused by single enzyme defects, and its use in dietary supplementation has been demonstrated to enhance cholesterol absorption in humans [35]. Additionally, a pilot study in 12 participants with SLOS who were treated with CA demonstrated significant increases in plasma cholesterol in the majority of patients, providing proof of principle for this therapeutic strategy [7].

To our knowledge, this is the first reported series of patient cases describing the real-world application of CA in combination with cholesterol supplementation for the treatment of SLOS. Importantly, the cases included in this series demonstrate the diversity of phenotypes and the variable nature of disabilities found in untreated patients with SLOS, including behavioral problems, inability to communicate, cognitive impairment, and challenges pertaining to activities of daily living. In aggregate, the cases provide valuable insights because they allow for assessment of the global impact of treatment across several disease manifestations as well as assessment of longer-term impact of treatment. Here we summarize some of the more salient learnings.

**Fig. 4:**
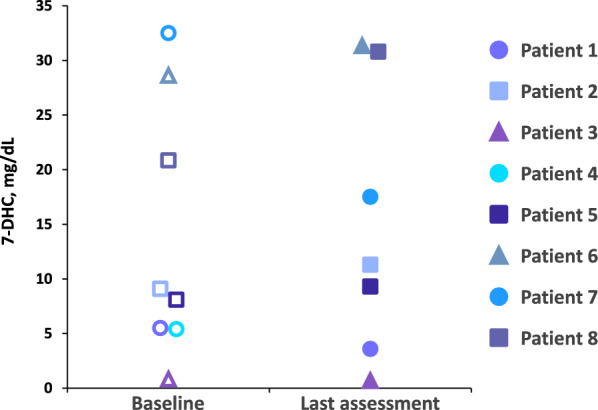
7-DHC levels for individual patients at baseline^a^ and at last assessment^b^ following treatment with cholic acid. Each patient is represented by a unique symbol and color. Open symbols designate baseline 7-DHC levels; closed symbols indicate 7-DHC levels at last assessment. ^a^Baseline for each patient was defined as Day 0. ^b^Time of last assessment during treatment for each patient

**Fig. 5:**
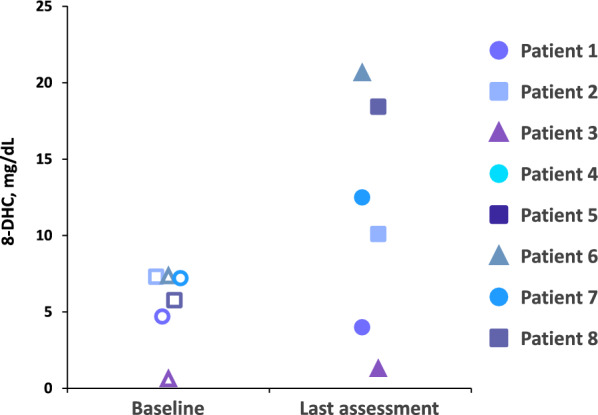
8-DHC levels for individual patients at baseline^a^ and at last assessment^b^ following treatment with cholic acid. Each patient is represented by a unique symbol and color. Open symbols designate baseline 8-DHC levels; closed symbols indicate 8-DHC levels at last assessment. ^a^Baseline for each patient was defined as Day 0. ^b^Time of last assessment during treatment for each patient

An initial key finding was the results of quantitative laboratory analyses. Despite variability in patient phenotypes and the noncentralized nature of the laboratory measurements, increases in total cholesterol over time were observed in seven of the eight patients following treatment with CA (Fig. 1), indicating that the medication has a positive impact on the underlying biochemical dysfunction. Patients with SLOS who have cholesterol levels ≥ 100 mg/dL are generally considered to be mildly affected. In this real-world retrospective case review, the majority of the patients had cholesterol levels ≥ 100 mg/dL. Even within this “mild” biochemical phenotype, objective improvements in cholesterol and growth and subjective improvements in behavioral/cognitive development were observed in the majority of these patients. Notably, variable effects on levels of 7-DHC and 8-DHC were observed following treatment with CA, with some patients showing substantial reductions over time and others showing increased levels despite overall increases in total cholesterol (Figs. 4 and 5). The reasons for these variable results are not clear, but it is possible that dysfunction of other pathways could affect production or metabolism of cholesterol precursors. Additional research may be warranted to elucidate the role of combining a statin with cholesterol supplementation and CA. This approach may provide a more responsive therapeutic clue to synergistically treating patients with SLOS to increase cholesterol while further downregulating toxic cholesterol precursor production by inhibiting HMG-CoA reductase proximal to the DHCR7 defect.

A second important finding was the consistency of improvements in patient behavior, QoL, cognitive ability, physical development, and/or physical functioning following treatment with CA (Table 2). Because one patient was only 0 to 12 months of age during the study period, QoL was assessed in seven patients, with positive changes in QoL in seven patients and positive behavioral changes in six patients. It is noteworthy that, in many instances, these significant behavioral and developmental improvements were observed after only a short time on treatment and subjectively reported by family members. Moreover, substantial subjective improvements in cognition, physical status, and QoL were observed in Patient 6 despite receiving treatment as an adult, with behavioral decline observed upon temporary discontinuation of treatment. Additionally, pretreatment and posttreatment height and weight Z-scores were available for 5 patients, with improvements observed in 4 of 5 patients for each of the parameters (Figs. 2 and 3). These data suggest that CA may have a direct and rapid impact on the most debilitating sequelae of SLOS, some of which are irreversible in the absence of effective intervention, even within the context of highly diverse phenotypes of the disease.

A final finding with potentially broad implications for treatment of patients with SLOS concerns the overall clinical benefit observed with CA plus cholesterol supplementation. Notably, improvements in laboratory parameters as well as behavior and development were observed both in patients who received treatment for ≤ 6 months (see available data for Patients 4, 5, and 7) and in those who received treatment for > 6 months (see available data for Patients 1, 2, 3, 6, and 8). Moreover, some patients were on therapy over a period of 350–450 days with durable benefit observed. Cholic acid supplementation has a well-established safety profile, with minimal side effects associated with the administration of this exogenous bile acid. In this case series report, there was one patient who had “transient” color change in the fingers and toes that was considered treatment related. Additionally, minimal changes in liver enzymes were observed during the corresponding treatment periods. No other associated adverse events were noted.

A limitation of this analysis is that only a small number of patients were included, and not all disease phenotypes are represented, which has the potential to skew some of the observed treatment responses. Additionally, because patients were treated in a real-world setting and not as part of a structured, controlled trial, there was variability in the timing of diagnosis, documentation of baseline characteristics such as height and weight, and reporting of laboratory parameters as well as variable patterns of clinical management, including the timing and type of cholesterol supplementation used, dosing of CA, duration of treatment with CA, and reporting of treatment outcomes. Consequently, the results and their broader applicability should be interpreted with caution. Importantly, Elias et al. 2024 addresses CA use in SLOS [7]. They show data for 2 months only. The data on CA use in SLOS are very limited and their study was the first to evaluate CA treatment in SLOS for various parameters. Here, we show data after CA treatment for as early as 1 month up to 450 days. While there is variability in assessing across patients, we focus on interpretation of the data within each patient separately (ie, each patient is acting as their own control). An additional limitation of this real-world case series is that bile acids were not measured in these patients. As mentioned earlier, the majority of the patients in this case series had cholesterol levels ≥ 100 mg/dL, which is generally associated with a milder biochemical phenotype. However, higher plasma cholesterol does not guarantee normal bile acid synthesis. It is known that cholesterol absorption in SLOS is decreased, and that decreased absorption is almost certainly due to bile acid deficiency, as bile acids are critically important in cholesterol absorption [36]. Decreased absorption was noted in that study despite most patients having cholesterol levels > 100 mg/dL. Furthermore, the pilot study from Elias et al. 2024, found that CA supplementation increased plasma cholesterol in most patients with SLOS, even in those with relatively high baseline levels (three of the 12 patients in that study had baseline cholesterol levels of approximately 100 mg/dL or higher). In addition, bile acid synthesis does not appear to correlate with baseline cholesterol levels in SLOS [37]*.* Taken together, available data then suggest that baseline cholesterol levels alone are not a reliable predictor of bile acid sufficiency. Furthermore, the cognitive, developmental, and QoL assessments were not standardized across institutions. Every institution asked questions of the patient and their caregiver (and included the treating physician’s observations about the patient) in a manner consistent with their institution. While there is variability across the patients in how these qualitative assessments were measured, the purpose of this study was to collectively assess these parameters for eight independent patients using their baseline values as their own control. However, despite these limitations and the diversity of clinical history, there was a remarkable concordance between treatment with CA and objective improvements in laboratory assessments as well as subjective behavioral, developmental, and QoL measures. Specifically, increases in total cholesterol levels and measurable improvements in cognitive/physical function and behavioral/QoL parameters were observed in nearly all cases following treatment. However, it is important to note that since systematic testing utilizing cognitive, developmental, and behavioral validated assessment tools was not undertaken in this case series, caution should be used in overinterpretation of these data. Because these benefits were observed across the spectrum of disease phenotypes represented by the diverse cases in this series, the findings suggest the potential for long-term management of these patients, and they support further evaluation of CA as a treatment option for patients with SLOS. Future research utilizing a long-term controlled trial approach with validated cognitive/behavioral assessments along with systematic evaluation of bile acid levels will be important in order to assess the impact of CA treatment in this patient population.

## Data Availability

Beginning 6 months and ending 5 years after publication, deidentified participant data might be made available to investigators whose proposed use of the data has been approved by a review committee, including the primary authors and the study funder. Proposals should be directed to grants@mirumpharma.com. Before being granted access, data requesters will be required to sign a data access agreement.
